# Personality interacts with habitat quality to govern individual mortality and dispersal patterns

**DOI:** 10.1002/ece3.4257

**Published:** 2018-06-22

**Authors:** Benjamin A. Belgrad, Blaine D. Griffen

**Affiliations:** ^1^ Marine Science Program School of Earth, Ocean, and Environment University of South Carolina Columbia South Carolina; ^2^ Department of Biology Brigham Young University Provo Utah

**Keywords:** dispersal, individual variation, *Panopeus herbstii*, predation risk, predator avoidance behavior

## Abstract

Individual phenotypic differences are increasingly recognized as key drivers of ecological processes. However, studies examining the relative importance of these differences in comparison with environmental factors or how individual phenotype interacts across different environmental contexts remain lacking. We performed two field experiments to assess the concurrent roles of personality differences and habitat quality in mediating individual mortality and dispersal. We quantified the predator avoidance response of mud crabs, *Panopeus herbstii*, collected from low‐ and high‐quality oyster reefs and measured crab loss in a caging experiment. We simultaneously measured the distance crabs traveled as well as the stability of personalities across reef quality in a separate reciprocal transplant experiment. Habitat quality was the primary determinant of crab loss, although the distance crabs traveled was governed by personality which interacted with habitat quality to control the fate of crabs. Here, crabs on low‐quality reefs rapidly emigrated, starting with the boldest individuals, and experienced modest levels of predation regardless of personality. In contrast, both bold and shy crabs would remain on high‐quality reefs for months where bolder individuals experienced higher predation risk. These findings suggest that personalities could produce vastly different population dynamics across habitat quality and govern community responses to habitat degradation.

## 1 INTRODUCTION

Individual mortality and dispersal rates are fundamental drivers of population and community dynamics as these life history traits can influence such processes as gene flow, species’ distribution, population growth, and inter/intraspecies encounter rates (Bowler & Benton, 2005; Clobert, Danchin, Dhondt & Nichols, 2001; Harwood & Hall, 1990; McPeek & Peckarsky, 1998). Both mortality and altered dispersal are also common responses to environmental changes (e.g., habitat destruction, Cushman, 2006; ocean acidification, Nagelkerken & Munday, 2016; seasonal change, Carlson, Olsen & Vøllestad, 2008; climatic regime shifts, Robinson et al., 2009). Understanding the factors which govern the mortality and dispersal responses to these global changes is therefore critical for managing populations and predicting the persistence of species in the face of these disturbances. For decades, scientists have recognized the importance of phenotypic differences in mediating mortality and dispersal rates (Clobert et al., 2001; Johnson, Grorud‐Colvert, Sponaugle & Semmens, 2014). While the majority of studies on phenotype‐dependent mortality and dispersal have focused on the effects of size (e.g., Skibinski & Roderick, 1991; Verhulst et al., 1997), age (Bradley, Wooller, Skira & Serventy, 1989; Hake, Kjellén & Alerstam, 2003), physiology (Ketterson & Nolan, 1992; Zera & Denno, 1997), and morphology (Koumoundouros, Maingot, Divanach & Kentouri, 2002; Lockwood, Swaddle & Rayner, 1998) are also commonly examined traits. One characteristic which has recently gained recognition as a crucial parameter within community interactions and as an important factor in determining mortality and dispersal rates is animal personality (Belgrad & Griffen, 2016; Cote, Clobert, Brodin, Fogarty & Sih, 2010; Dingemanse & Wolf, 2010; Pennisi, 2016).

Animal personalities, defined as consistent differences in individual behavior over time or context, have been documented across a broad range of phyla (Freeman & Gosling, 2010; Gosling, 2001; Stamps & Groothuis, 2010) and have strong individual, population, and community‐level impacts (cataloged in Wolf & Weissing, 2012). For instance, personalities can govern life history traits and fitness through differential use of the local environment as exploratory behavior in songbirds was found to influence fecundity (Both, Dingemanse, Drent & Tinbergen, 2005; Dingemanse, Both, van Noordwijk, Rutten & Drent, 2003; Stamps, 2007). Furthermore, personalities can shape community dynamics by cascading effects derived from distinctive species interactions (Belgrad & Griffen, 2016; Griffen, Toscano & Gatto, 2012; Moran, Wong & Thompson, 2017) as well as through differential dispersal patterns (Cote & Clobert, 2007) and disease spread (Krause et al., 2010). For example, bold mud crabs consume more prey (Griffen et al., 2012) and are preferentially consumed by different predators than shy mud crabs (Belgrad & Griffen, 2016) which can potentially control bivalve recruitment on oyster reefs. Such behavioral variability has also been suggested to mediate invasion success (Carere & Gherardi, 2013) and speciation (Wolf & Weissing, 2012).

Despite widespread acknowledgment that animal personalities and other individual phenotypic differences play an important role in community processes, our knowledge of the relative importance of these differences in comparison with environmental parameters or how individual phenotype interacts across environmental gradients, such as habitat quality, remains limited (Toscano, Gownaris, Heerhartz & Monaco, 2016). This is exacerbated by a dearth of field‐based personality studies that span different contexts (Dall & Griffith, 2014; Wolf & Weissing, 2012). A number of studies have directly linked individual boldness and decreased refuge use to increased mortality (e.g., Belgrad & Griffen, 2016; Bremner‐Harrison, Prodohl & Elwood, 2004) and dispersal distance (e.g., Cote, Fogarty, Brodin, Weinersmith & Sih, 2011; Dingemanse et al., 2003). However, the opposite trend in mortality has also been found in some instances as the relationship can depend on the predator encountered (e.g., Réale & Festa‐Bianchet, 2003; Smith & Blumstein, 2010). Additionally, decreases in habitat quality have frequently been associated with elevated mortality (Cushman, 2006; Rodwell, Barbier, Roberts & McClanahan, 2003), mass emigrations (Lenihan et al., 2001; Matter & Roland, 2002), and altered movement patterns (Bélanger & Rodríguez, 2002). As habitat degradation commonly involves a loss in refuge availability, there is a high potential for interactions between personality and habitat quality to exist.

Furthermore, our understanding of the potential interaction between personality and habitat quality is further constrained by the lack of empirical studies on the persistence of personalities (Cote et al., 2010). While personalities were traditionally viewed as stable and behaviorally limiting, recent theory suggests individuals can show considerable behavioral plasticity across contexts and may alter their behavior in response to dramatic changes in environmental conditions (i.e., behavioral reaction norms; Dingemanse, Kazem, Réale & Wright, 2010; Dingemanse & Wolf, 2013). Here, we conduct two mark–recapture field experiments to examine the interactive roles that personality and habitat quality play in governing individual mortality, dispersal, and behavioral stability within a model study system.

We conducted a caging experiment to test the effect of personality and habitat quality on individual mortality. Bolder individuals frequently forego refuge in the presence of predators (e.g., Belgrad & Griffen, 2017) so individuals exhibiting bold behaviors likely will experience higher mortality than shy individuals. However, differences in mortality may decrease with decreasing habitat quality as refuges become scarce in low‐quality habitat and shyer individuals become preyed upon. Alternatively, mortality differences between personality types may actually increase as bold individuals become even further exposed. We also conducted a reciprocal transplant experiment to test the effect of personality and habitat quality on individual dispersal and behavioral stability. Bold individuals are expected to disperse farther than shy individuals as boldness is generally associated with more mobility (e.g., Cote et al., 2011; Dingemanse et al., 2003). Dispersal differences between personality types are expected to be greatest in low‐quality habitats as individuals should travel more in an effort to seek better quality habitat. Personality types are expected to be stable when individuals remain in the same habitat. However, individuals transplanted to high‐quality habitats may become bolder due to an increase in food and refuge availability, while individuals transplanted to low‐quality habitat may become shyer as these resources decrease.

## 2 MATERIALS AND METHODS

### 2.1 Study system

We conducted our experiments in a coastal habitat which is spatially heterogeneous at small scales (<100 m). Found worldwide, oyster reefs can be a dominant commercial and ecological component of estuaries, but have declined by 85% globally (Beck et al., 2011) due to a variety of anthropogenic sources including harvesting, sedimentation, disease, introduced pests, and oxygen depletion (Lenihan & Peterson, 1998). The degradation of oyster reefs produces a gradient of high‐ to low‐quality habitat and has ramifications beyond the oysters themselves, as these organisms are considered ecosystem engineers that provide valuable shelter and food for a diverse array of species (Lenihan & Peterson, 1998; Lenihan et al., 2001).

One such species is the mud crab, *Panopeus herbstii*, which is a common consumer within reefs along the Atlantic and Gulf coasts of the United States (Grabowski, 2004; McDonald, 1982). The mud crab is relatively stationary compared to other crab species which use the same habitat, as individual mud crabs can stay on the same reef for months (Toscano, Gato & Griffen, 2014), although some may travel >5 m over 2 days (Stachowicz & Hay, 1999). Crab movement is primarily attributed to foraging upon bivalves such as juvenile oysters, *Crassostrea virginica*, and the scorched mussel *Brachidontes exustus* (Toscano & Griffen, 2012). However, crab movement can also comprise mate searching and brief competitive interactions (Belgrad, personal observations). Individuals greatly reduce their activity levels and increase their time in refuge in the presence of predators (Hughes, Mann & Kimbro, 2014), exhibiting a continuum of bold—shy personalities. Previous work in this system has established that individual *P. herbstii* exhibit just such persistent personality differences. Specifically, differences in activity level and in refuge use between individuals on the same reef, reflecting personality, can last for months (Toscano et al., 2014) and are consistent across a range of different conditions, including predator presence/absence (Griffen et al., 2012), conspecific density changes (Belgrad & Griffen, 2017), and starvation levels (B. A. Belgrad 2015, unpublished data).

We conducted our study during the peak and mid spawning season of crabs on oyster reefs within the North Inlet National Estuarine Research Reserve in South Carolina as these time periods are when crabs are the most active (McDonald, 1982). This estuary supports a mixture of low‐ and high‐quality reefs that cover extensive areas within intertidal channels.

### 2.2 Effects of personality and reef quality on predation risk

We conducted a 2‐week caging experiment once during May and again during July 2016 to determine how personality and habitat quality simultaneously govern individual mortality and movement in the field. Four low‐quality and four high‐quality reefs were sampled within North Inlet. Reef quality was determined by reef height as oyster survival and food availability strongly correlate with this parameter (Lenihan & Peterson, 1998), as well as rugosity and oyster density (unpublished data). Low‐quality reefs were defined as reefs with heights less than 10 cm, while high‐quality reefs had heights greater than 20 cm. Reef height was calculated following procedures described in Griffen and Norelli (2015).

Within each reef, we set up three haphazardly placed 2.5 × 2.5 m plots that were spaced at least 5 m apart. A vexar mesh cage (pore size = 3.2 cm, height = 1.0 m) completely enclosed one plot to exclude predators, but allowed crabs to move freely out of the cage. Rebar (width = 1.9 cm, length = 2 m) and tent stakes (Coleman, length = 25.4 cm) were arrayed around the cage perimeter to keep the mesh edges buried ~3 cm into the sediment. No predatory toadfish, stone crabs, or blue crabs were found to have invaded the cages while sampling. The second plot only had two opposite sides staked with vexar mesh and no mesh top to control for the effects of caging. The final plot did not have any mesh to maintain natural reef conditions. Ten mature mud crabs (mean ± *SD* carapace width = 25.1 ± 1.9 mm; 204 males, 276 females) were collected by hand from each plot. Cohorts collected from the same plot were kept together throughout the entire study. Crab collections were blocked in time with two high‐ and two low‐quality reefs sampled on 1 day and the remaining four reefs sampled the next day (*n* = 4 reefs of each quality). Collected crabs were brought to the Baruch Institute wet laboratory to assess individual personality.

Personality was assayed in natural cohorts following an established protocol (for a detailed description see Belgrad & Griffen, 2016). Briefly, cohorts were starved for 24 hr to standardize hunger levels, marked with nontoxic nail polish (100% Pure, San Jose, California, USA), and placed inside separate flow‐through mesocosms (circular with diameter 1 m; water height 15 cm). Crabs from both high‐quality and low‐quality reefs were subjected to a common garden experiment where individuals were exposed to oyster clumps of intermediate shell density and height, relative to our field‐sampled reefs, with ample structure to provide refuge, and with predator and prey odor cues delivered continuously. We conducted all observations at night under red light to ensure mud crabs were at their most active and were undisturbed by the observer. Crabs were given 10 min to acclimate once cohorts were placed in the mesocosms. After acclimating, we recorded whether each individually marked crab was exposed on the shell surface layer or hiding underneath the oysters every 9 min for 3 hr (20 observations for each crab). Refuge use was measured as the proportion of these 20 observations in which crabs were in refuge and not visible to the observer in the same manner used in prior studies of mud crab behavior (Belgrad & Griffen, 2016; Belgrad, Karan & Griffen, 2017; Griffen et al., 2012; Toscano et al., 2014).

Following behavioral observations, crabs were marked more permanently with individually numbered bee tags (the Bee Works, Orillia, Ontario, Canada) and released to the same plot from which they were collected. Every 48 hr for 2 weeks each plot was exhaustively surveyed by hand during low tide to determine which individuals remained in the plots. Individuals not found within the completely enclosed plots were assumed to have emigrated from the region, while those not found in the open plots may have either emigrated or been consumed by predators. Following the 2‐week survey period, new plots were established in different sections of each reef, and the experiment was repeated 1.5 months later to assess the consistency of our findings.

We evaluated how time to crab disappearance from plots was influenced by the fixed effects of caging treatment, reef quality, crab refuge use (i.e., personality measured in the laboratory), carapace width, gender, and month sampled using a mixed‐effects Cox proportional hazards model (i.e., a survival analysis). Reef ID and day sampled were treated as random effects to control for repeated measures on the same reef and variables associated with sampling time (R package: frailtyHL). A Cox proportional hazards analysis is a statistical model which distinguishes between maximum values that represents a specific event occurring and those that simply represent the end of the observational period, then ranks the data accordingly (i.e., the data are right censored). This model therefore allowed us to right censor the data to account for crabs that were still found in our plots on the last day of the survey. This analysis was conducted using R v3.2.3 (R Foundation for Statistical Computing, Vienna, Austria).

As several crabs were not spotted during some inspection periods but would reappear in later inspection periods, we estimated the parameters apparent survival (*ϕ*) and resighting probabilities (*p*) simultaneously using the program MARK (White & Burnham, 1999). The overall dataset was analyzed with live recapture Cormack–Jolly–Seber (CJS) models using time‐dependent survival/recapture probabilities and a logit link function (Cooch & White, 2017). Here, CJS models use encounter data across multiple sampling efforts to calculate *ϕ* and *p* where the probability of encountering a tagged individual (*E*) equals the product of apparent survival and resighting probabilities for that particular sampling effort (i.e., *E*_*i*_ = *ϕ*_*i*_ *p*_*i*_) Parameter estimates were obtained from the model using numerical maximum likelihood. Average survival and recapture probabilities were calculated for each caging treatment, reef quality, month sampled, gender, bold (exposed 70%–100% of time), moderate (45%–65%), and shy crabs (0%–40%), while carapace width was treated as a cofactor. MARK analyzed each grouping individually from the full dataset. Personality groupings were chosen so that the number of crabs in each category was more evenly distributed as there was a preponderance of bold individuals.

### 2.3 Effects of personality and reef quality on dispersal

We simultaneously conducted a reciprocal transplant experiment between May and August 2016 to evaluate the roles that individual personality and habitat quality play in governing crab dispersal over extended timeframes under natural conditions. An additional six high‐quality and six low‐quality reefs were identified in the inlet. Three high‐ and three low‐quality reefs were randomly designated as transplant reefs, while the remaining six reefs were controls. Twenty mature crabs (mean ± *SD* carapace width = 24.8 ± 1.8 mm; 135 males, 105 females) were collected from each reef. Crabs were collected by hand in cohorts of 10 individuals from 1 m^2^ plots (two cohorts per reef). Sampling area was widened if 10 crabs were not found within the plots in an effort to maintain crab densities during behavior measurements and ensure each treatment had an equal number of crabs. Collections were blocked through time as each consecutive day one high‐ and one low‐quality reefs were sampled. We transported the crabs to the Baruch laboratory where their individual personality was assayed in the same manner as the previous experiment.

Within 24 hr of quantifying personality, crabs were marked with numbered aluminum tags (diameter = 12.7 mm; The Tag Place). Crabs collected from transplant reefs were returned to reefs of the opposite quality, while crabs collected from control reefs were returned to their original reefs. Numbered stakes were placed where crabs were released on each reef. Seven, 45, and 90 days after crabs were released, reefs were surveyed at low tide in a 25 m radius from the release points with a metal detector (Tesoro Sand Shark) and by hand to recapture crabs. Distance migrated from the stakes was measured during each survey. Crabs located at the day 7 survey were left undisturbed, while crabs found 45 and 90 days after their release were brought back to the laboratory and had their personality reassessed in the same manner as before to determine the extent that their personality changed over time. As not all crabs were recaptured for the 45‐ and 90‐day behavior assays, and to help prevent behavioral changes due to differences in conspecific density, supplementary crabs were caught from the same reefs to ensure that behavior continued to be measured in cohorts of 10. Crabs were again released 24 hr after measuring behavior to either their transplant or control reefs depending on their treatment.

Given the large number of crabs that were not recaptured, recapture success and duration on reefs were analyzed with zero‐inflated mixed‐effects generalized linear models using a binomial and Poisson distribution, respectively (GLMs; R package: glmmADMB). We treated transplant treatment, reef quality, crab refuge use, carapace width, and gender as fixed effects, and reef ID as well as day collected as random effects. Both the maximum distance crabs traveled and the distance traveled in the first week were analyzed with standard mixed‐effects GLMs in the same manner as above using only recaptured crabs (R package: lme4). We were unable to statistically analyze crab behavior changes due to vastly uneven recapture success across treatments. We discuss the trends in this data below. MARK analyses were not conducted for this experiment as all resighted crabs were observed during each previous inspection.

## 3 RESULTS

### 3.1 Effects of personality and reef quality on predation risk

**Figure 1:**
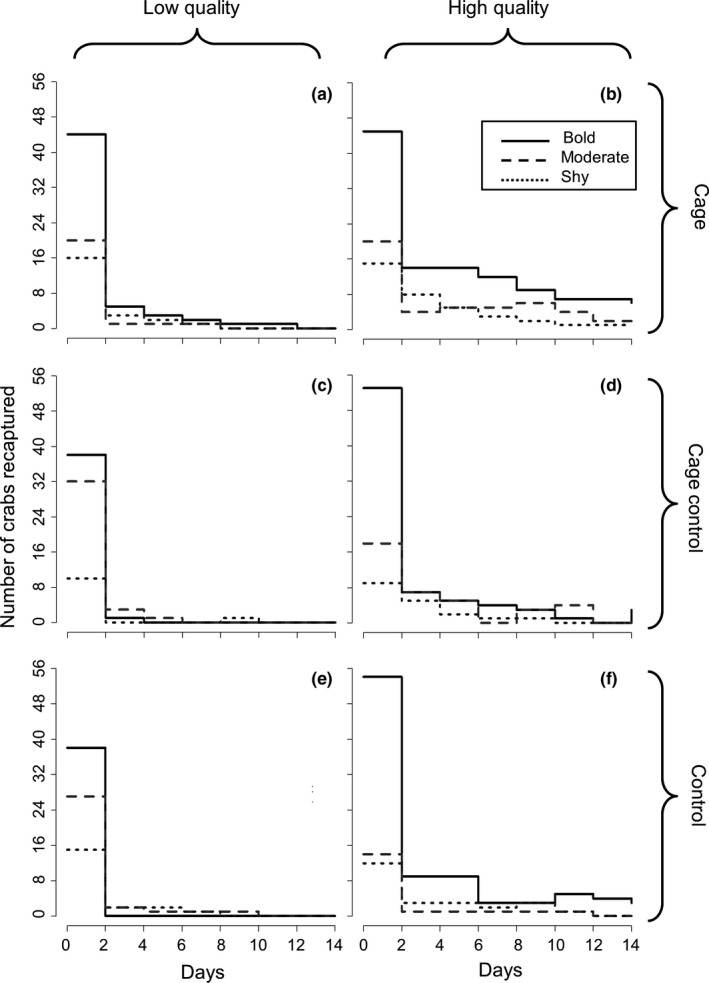
Number of bold (exposed 70%–100% of time), moderate (45%–65%), and shy crabs (0%–40%) that remained within (a,b) completely enclosed, (c,d) partially enclosed, and (e,f) open 2.5 m^2^ plots on (a,c,e) low‐quality and (b,d,f) high‐quality reefs over 14 days (*n* = 8 reefs total; 80 crabs per treatment). Day zero shows the initial personality distribution of crabs within each treatment. Increases in the step functions indicate crabs which left the plot 1 day and returned at a later sampling date

Predation risk was heavily dependent on both the external environment and individual characteristics as reef quality, caging treatment, and individual personality significantly interacted to govern crab survival and recapture probabilities (Table 1). Overall, low‐quality plots had 44% more crabs disappear within 48 hr than high‐quality plots, and no crabs remained in low‐quality plots throughout the entire experiment, while 15 crabs stayed in high‐quality plots the entire time (Figure 1). Consequently, crabs on high‐quality plots had a 23% higher survival probability and 74% greater recapture success than crabs on low‐quality plots throughout the entire experiment (Table 2). Crabs in caged treatments had, on average, 90% higher survival probability than noncaged treatments and virtually no difference in recapture success. Bold crabs (exposed 70%–100% of the time) had 18% lower survival probability than moderate crabs (exposed 45%–65% of the time) and 28% lower survival probability than shy crabs (exposed 0%–40% of the time). In contrast, the recapture success of bold crabs was ~13% higher than moderate or shy crabs which was a result of bold crabs remaining for extended periods of time in caged plots on high‐quality reefs (Table 2; Figure 1).

**Table 1:** Descriptive statistics of a mixed‐effects Cox proportional hazards model examining the impact of reef quality, carapace width, caging treatment, personality, month sampled, and gender on *Panopeus herbstii* recapture success

Fixed effect	Estimate	*SE*	*Z*	*p*
Reef quality	−0.94	0.45	−1.99	0.047
Carapace width	0.06	0.02	2.99	0.003
Caging treatment	−0.55	0.31	1.78	0.076
Personality	0.24	0.26	0.91	0.363
Month sampled	−0.01	0.27	−0.02	0.982
Gender	0.02	0.08	0.29	0.770
Caging treatment × reef quality	1.80	0.61	2.94	0.003
Personality × reef quality	−1.77	0.60	−2.95	0.003
Personality × caging treatment × reef quality	−3.85	1.13	−3.40	0.001

**Table 2:** Estimated survival and recapture probabilities of *Panopeus herbstii* grouped by reef quality, caging treatment, personality, month sampled, and gender

Group	Apparent survival probability			Recapture probability		
	Estimate	*SE*	CI (95%)	Estimate	*SE*	CI (95%)
High‐quality reef	0.62	0.07	0.48–0.75	0.72	0.09	0.43–0.82
Low‐quality reef	0.51	3.42	0.25–0.91	0.41	4.90	0.21–0.73
Cage treatment	0.80	1.93	0.36–0.91	0.71	1.88	0.38–0.84
Cage control treatment	0.35	2.18	0.12–0.69	0.70	3.61	0.06–0.90
Control treatment	0.56	1.42	0.14–0.76	0.69	1.47	0.05–0.83
Bold	0.60	0.08	0.43–0.75	0.71	0.11	0.40–0.84
Moderate	0.71	3.45	0.16–0.87	0.64	3.17	0.12–0.81
Shy	0.77	0.11	0.16–0.93	0.62	0.22	0.13–0.84
First sampling period	0.78	11.53	0.34–0.90	0.67	11.20	0.28–0.82
Second sampling period	0.50	0.51	0.20–0.69	0.68	0.52	0.18–0.84
Male	0.69	1.99	0.30–0.87	0.65	1.98	0.18–0.81
Female	0.61	0.57	0.30–0.76	0.69	0.60	0.27–0.84

*Note*

Estimates obtained from live recapture Cormack–Jolly–Seber (CJS) models.

Indeed, crab retention on reefs depended on multiple interactions between reef quality, caging treatment, and personality (Table 1). Caging treatment generally had a larger effect on low‐quality reefs than in high‐quality reefs. On low‐quality reefs, double the crabs were found at least once in completely enclosed plots compared to the partially caged and open plots (Figure 1a,c,e), whereas on high‐quality reefs, only ~56% more crabs were found in completely enclosed plots than in the other two treatments (Figure 1b,d,f). Additionally, personality had a greater impact on crab retention in high‐quality reefs than in low‐quality reefs, and differences between crab retention times were magnified across reef quality. While shy crabs on average were found within high‐quality reefs 3× longer than on low‐quality reefs, moderate crabs were found within high‐quality reefs for ~7× longer, and bold crabs remained on high‐quality reefs >12× longer than on low‐quality reefs. The increased recapture success of bold crabs on high‐quality reefs was particularly notable in the completely enclosed cage treatment. Bold crabs were found almost twice as long on average in completely enclosed plots (Figure 1b) than in the partially caged (Figure 1d) and open plots (Figure 1f). Furthermore, while all crabs were found the most frequently in completely enclosed plots for both high‐ and low‐quality reefs, only in high‐quality reefs were bold crabs found more often in the plots than shy or moderate crabs.

Individual size also had an impact on crab retention as larger crabs were found on plots significantly longer than small crabs even though the parameter effect size was small (Supporting Information Figure S1, Table 1). Neither month sampled nor gender had a significant impact on whether crabs were found (Table 1) as recapture probabilities remained similar between treatments. However, crab survival probability was 35% lower during the second sampling period (Table 2).

### 3.2 Effects of personality and reef quality on dispersal

**Figure 2:**
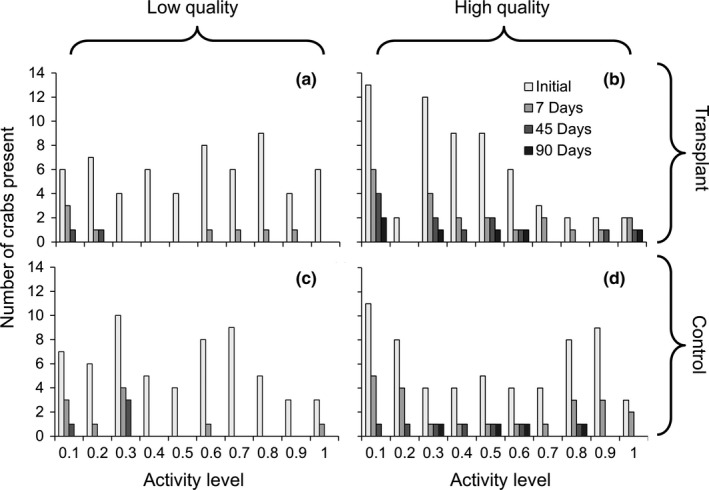
Number of crabs found within (a,c) low‐ and (b,d) high‐quality reefs according to their initial activity level 7, 45, and 90 days after their release (*n* = 12 reefs total). Crabs were either (a,b) transplanted to reefs of the opposite quality or (c,d) originated on those reefs (control; *n* = 60 crabs per treatment). Activity level was measured as the proportion of time crabs spent active outside of refuge over 3 hr

**Figure 3:**
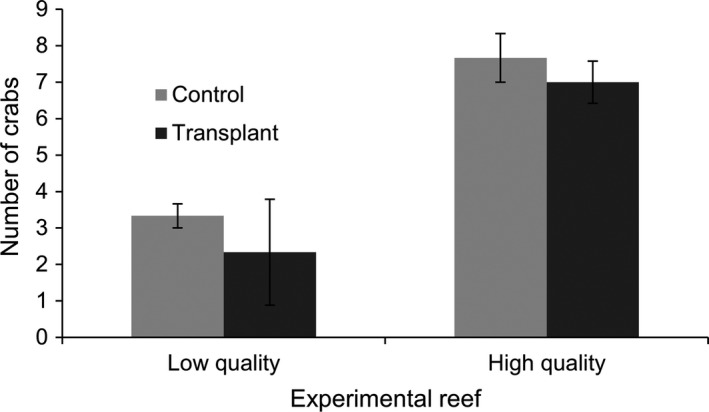
Mean ± *SE* number of crabs recaptured from low‐ and high‐quality reefs (*n* = 12 reefs total) that were either transplanted to reefs of the opposite quality or originated on those reefs (control)

During the transplant experiment, 61 of 240 crabs were found 7 days after their release, which dropped to 25 and 10 individuals 45 and 90 days after their release (Figure 2). Crab retention on reefs was dependent on significant interactions between reef quality, transplant treatment, and individual personality (Table 3a). On average, ~2.5 times more crabs were recaptured on high‐quality than low‐quality reefs (Figure 3), and no crabs were found within low‐quality sampling sites after 3 months (Figure 2a,c). Significantly more crabs were recaptured when they were released within their original reef rather than when transplanted to a reef of the opposite quality (Table 3a). Approximately 4.8% more crabs were recaptured from high‐quality reefs if they originated on the reef rather than being transferred to the reef, while 25% more crabs were recaptured on low‐quality reefs if they originated on the reef than when transplanted (Figure 3) Similar to the caging experiment, bolder crabs generally had shorter retention times on reefs than shy individuals. However, this varied with reef quality as some moderate and bold crabs remaining on high‐quality reefs for at least 90 days (Figure 2b,d), while only a few shy individuals were found on low‐quality reefs 45 days after their release (Figure 2a,c). Interestingly, bold crabs transplanted to new reefs were recaptured more than bold individuals which originated on high (66.6% transplant vs. 37.5% original) and low‐quality reefs (12.0% transplant vs. 5.0% original), whereas shy crabs which were transplanted to a new reef were recaptured less than shy crabs which originated on high‐ (33.3% transplant vs. 40.7% original) and low‐quality reefs (17.4% transplant vs. 28.6% original; Figure 2).

**Table 3:** Descriptive statistics of zero‐inflated mixed‐effects generalized linear models examining the impact of reef quality, transplant treatment, personality, month sampled, gender, and carapace width on (a) initial *Panopeus herbstii* recapture success and (b) distance traveled

Fixed effect	(a) Duration on reefs				(b) Distance traveled			
	Estimate	*SE*	*Z*	*p*	Estimate	*SE*	*Z*	*p*
Transplant	1.57	0.59	2.68	0.008	−0.02	0.24	−0.07	0.945
Personality	0.02	0.32	0.02	0.983	0.12	0.05	2.72	0.007
Reef quality	−0.13	0.28	−0.16	0.869	−0.05	0.24	−0.21	0.838
Gender	<0.01	0.15	0.01	0.991	0.47	0.02	27.70	<0.001
Carapace width	0.01	0.04	0.16	0.876	0.15	<0.01	29.49	<0.001
Personality × transplant	0.01	0.49	0.01	0.989	0.35	0.12	3.02	0.003
Personality × reef quality	0.02	0.56	0.03	0.973	1.06	0.07	14.42	<0.001
Personality × reef quality × transplant	2.44	0.72	3.37	<0.001	−0.53	0.14	−3.94	<0.001

**Figure 4:**
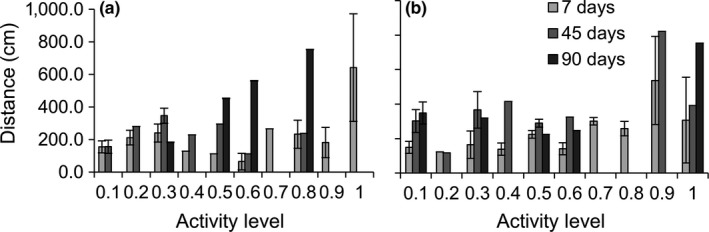
Mean ± *SE* distance crabs traveled (cm) from their release point according to their activity level when (a) released on their original reef or (b) transplanted to a reef of the opposite quality (*n* = 1–9 depending on the category). The absence of error bars indicates that only one individual was captured in that category

**Figure 5:**
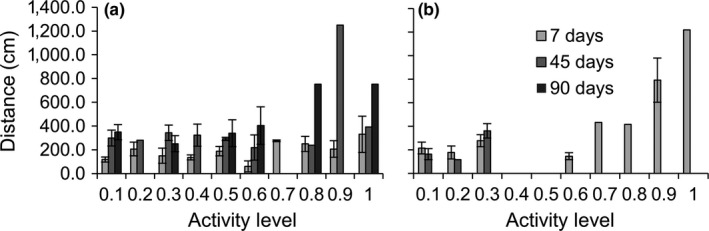
Mean ± *SE* distance crabs traveled (cm) from their release point according to their activity level when released on either (a) high‐quality or (b) low‐quality reefs (*n* = 1–11 depending on the category). The absence of error bars indicates that only one individual was captured in that category

**Figure 6:**
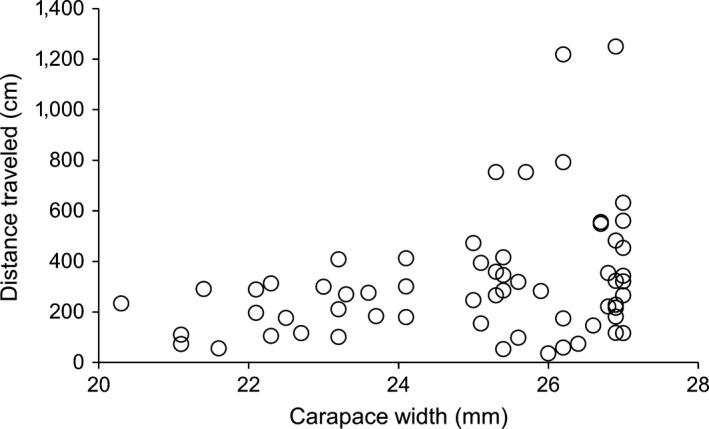
Relationship between crab carapace width (mm) and maximum distance traveled (cm) from their release point (*n* = 61)

Most crabs which remained on reefs were found within three meters of their release point, and some within half a meter (Figure 4). Individual dispersal distance depended on a significant three‐way interaction between reef quality, transplant treatment, and personality (Table 3b). Bold crabs traveled farther than shy crabs, especially as time passed, as recaptured shy crabs on average remained within three meters for the entire study, whereas recaptured bold crabs more than doubled this distance (Figures 4 and 5). These differences in distances are conservative as bold crabs were also 10% more likely to leave the sampling area later in the season. Crabs on their original reef increased their dispersal distance with boldness, while this trend was less pronounced in transplanted crabs (Figure 4b). Crabs in low‐quality reefs had a strong positive relationship between crab boldness and distance traveled, whereas this relationship was weaker on high‐quality reefs as bolder individuals traveled less on high‐quality reefs (Figure 5). Additionally, female crabs on average traveled 71% farther than males, and larger crabs traveled substantially farther than smaller individuals (Figure 6; Table 3b).

**Figure 7:**
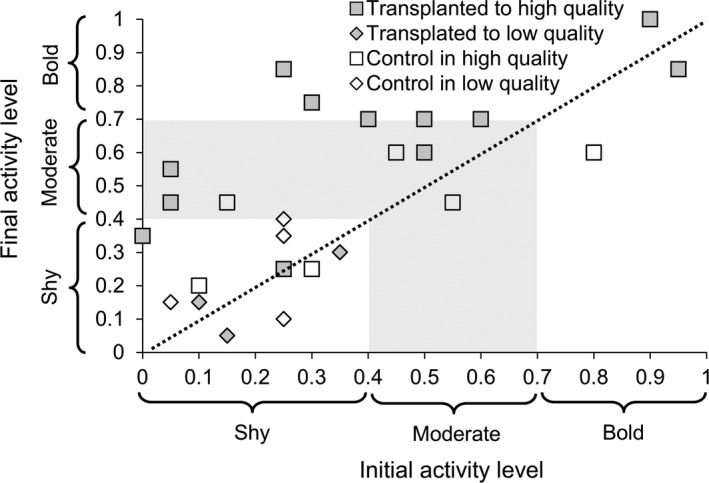
Individual *Panopeus herbstii* activity levels measured 1.5–3 months after their initial behavioral assay and subsequent release to their original reef (open symbols) or transplant to a reef of the opposite quality (filled symbols; high‐quality reef = diamond symbols, low‐quality reef = square symbols; returned to high‐quality reef *n* = 6, returned to low‐quality reef *n* = 4, transplanted to high‐quality reef *n* = 12, and transplanted to low‐quality reef *n* = 3). Dotted line indicates perfect behavioral consistency, while shaded region denotes borders of shy, moderate, and bold personalities

Finally, there were trends in behavioral changes among recaptured crabs, but this was not examined statistically because of the low number of crabs recaptured and the unequal sample sizes. While crabs which remained on their original reef did not display any major behavioral changes, regardless of reef quality, crabs transplanted to new reefs exhibited divergent behavioral alterations. Whereas crabs transplanted to low‐quality reefs slightly decreased in boldness, crabs transplanted to high‐quality reefs increased the proportion of time spent active by 67% (Figure 7).

## 4 DISCUSSION

We have demonstrated that personality interacts with habitat quality and can help predict predation risk and individual movement within the wild. Although habitat quality was the main predictor of crab recapture success, our data indicate that individual personality produces vastly different outcomes in each habitat type. Whereas high‐quality reefs were characterized by both bold and shy crabs remaining on the reefs for extended periods of time and moderate predation of bold individuals, our results suggest that low‐quality reefs had modest levels of predation of all personality types with the vast majority of crabs emigrating from the region immediately, starting with the boldest individuals. These results have important implications for numerous study systems and support conceptual theories on the role of personality in mediating community dynamics (Cote et al., 2010; Sih, Cote, Evans, Fogarty & Pruitt, 2012; Spiegel, Leu, Bull & Sih, 2017; Wolf & Weissing, 2012).

Our conclusions are derived by the similarities in crab loss rates between the completely caged and partially caged/open plots which suggest that the majority of crab loss is from emigration out of the plots, while the differences between treatments denote that predation risk is highest among bold individuals. Such findings are consistent with our previous laboratory predation study which found that bold crabs experience higher mortality (Belgrad & Griffen, 2016) and are further supported by the distances crabs traveled in the transplant experiment as well as the estimated crab survival/recapture probabilities (Table 2). Estimates of differences in crab predation risk are likely conservative as crabs in caged plots may have migrated out of the cage and been consumed. Crabs which we were unable to locate on low‐quality reefs during the transplant experiment were predominantly assumed to have migrated out of the search area given the low recapture probability of crabs in caged plots on low‐quality reefs relative to high‐quality plots. However, higher predation risk is also liable to be partially responsible given the greater difference in recapture success between caged and uncaged plots on low‐quality reefs. While some individuals may have simply been missed during sampling by burrowing below the limit of the metal detector, these cases were likely rare, as we also thoroughly sampled the regions by hand. Disturbance from resampling the reefs and handling crabs may have increased the likelihood of crabs emigrating from the region or their predation risk. However, even when our disturbance was the most frequent, crabs could regularly be discovered under the exact same oyster clumps they utilized previously, while changes to immediate predation risk were minimized by returning crabs to their oyster clumps during low tide when their aquatic predators were absent. Similar to a previous study on the same system using laboratory mesocosms (Grabowski, 2004) and parallel to many other field studies on habitat quality (Cushman, 2006; Lenihan et al., 2001; Lin & Batzli, 2001; Matter & Roland, 2002; Rodwell et al., 2003), we found that overall crab survival probability was lowest in low‐quality reefs. Our observed increase in total crab predation among low‐quality reefs likely stems from an absence of refuge availability exposing moderate and shy crabs to predation and highlights the importance of accounting for both individual and environmental characteristics simultaneously.

Notably, crabs from different quality reefs faced different fates depending on their size and personality. Large crabs on low‐quality reefs likely were found within plots longer than smaller crabs because their increased size helped protect them from predation, whereas this phenomenon was almost nonexistent on high‐quality reefs due to the abundance of oyster shell refuges which could hide smaller individuals (Supporting Information Figure S1). Additionally, bold crabs from low‐quality reefs typically left the region within a week and generally traveled farther than their counterparts on high‐quality reefs, whereas a few shy crabs remained for over a month. In contrast, bold crabs from high‐quality reefs seemed to have higher levels of predation, and individuals stayed on the reefs for several months regardless of personality type. These results suggest that inhabitants of high‐quality reefs are likely semipermanent residents, while most crabs found on low‐quality reefs are likely transients. This also suggests personality can cause individuals living within different quality habitats to exhibit distinctive community interactions. For example, the tendency of bold crabs to leave low‐quality reefs and stay on high‐quality reefs may encourage predators to follow the same pattern, as higher trophic‐level predators seem to predominantly feed upon bold crabs. In contrast, prey species on low‐quality reefs might inordinately benefit from having predominantly shy crabs remain on these reefs as *P. herbstii* consumption rates correlate to boldness (Griffen et al., 2012; Toscano & Griffen, 2014), and group personality composition has been found in some social species to be more important than group size in controlling foraging (Keiser & Pruitt, 2014; Michelena, Sibbald, Erhard & McLeod, 2009).

At the population level, our findings suggest personality can influence the distribution of individuals by governing both dispersal distance and propensity to disperse. Indeed, bold crabs tended to travel farther than shy crabs on both reef types and low‐quality reefs commonly house a higher proportion of shy crabs than high‐quality reefs (this study, Belgrad et al., 2017). Such results substantiate personality‐dependent simulations on the home range and distribution of individuals developed by Spiegel et al. (2017) that indicate dispersal propensity can mediate the size of home ranges and clustering of personality types. Furthermore, the increased propensity of crabs to disperse on low‐quality reefs corroborate dispersal models incorporating habitat quality (Taylor & Norris, 2007), and our study shows that the addition of personality can help explain instances of partial dispersal within populations. Comparable relationships between boldness and dispersal have also been seen in fish (Cote et al., 2011; Fraser, Gilliam, Daley, Le & Skalski, 2001), birds (Dingemanse et al., 2003), lizards (Cote & Clobert, 2007), and other crab species (Knotts & Griffen, 2016), but none studied the simultaneous effects of habitat quality or mortality. Interestingly, even though the dispersal behavior of bold crabs caused low‐quality reefs to have greater proportions of shy crabs than high‐quality reefs, increased predation pressure of bold crabs on high‐quality reefs can serve to dampen differences in personality distribution between reefs. Seasonal changes in predator density may therefore help explain fluctuations in the distribution of personalities that have previously been observed among individual reefs (Belgrad et al., 2017). Such considerations toward personality distributions are important because the personality composition of populations has been found to control population mating success (Sih & Watters, 2005), offspring dispersal (Cote et al., 2011), and disease transmission (Keiser, Howell, Pinter‐Wollman & Pruitt, 2016).

The differences in community interactions discussed above between habitat types likely persist through time given that crabs which remained on their original reef maintained their personality for months (Figure 7). An observation seen previously in this system (Toscano et al., 2014), but which had not been tested across different quality habitats. Although crabs transplanted to different quality reefs may have altered their behavior and consequently their predation risk/dispersal propensity, previous personality studies in this system which span different environmental contexts (e.g., predation risk and conspecific density) found that relative behavioral differences between individuals remain similar when crabs shift their behavior to match environmental circumstances (Belgrad & Griffen, 2017; Griffen et al., 2012). In fact, the behavioral changes observed in transplanted crabs suggest that these differences in community interactions may be magnified by habitat degradation and dispersal as bold individuals that migrate to high‐quality reefs should become bolder, while crabs that find themselves on degraded, low‐quality reefs should either migrate or become shyer. Fascinatingly, bold transplanted crabs also had a higher recapture probability than shy transplanted crabs, while the reverse scenario occurred when crabs were returned to their original reef (i.e., shy crabs were recaptured more). Changes in crab retention due to the transplant experiment may have occurred because crabs were unaccustomed to the change in reef quality or because crabs were simply moved to a new location. However, the differences in recapture success observed across personality indicate that bold crabs may drive shy individuals away from prime habitat when transplanted; whereas crabs returned to their original reef already have a hierarchy established with the local crab population where shyer individuals already lay claim to nearby refuges. These results provide mechanisms through which populations may develop spatially explicit personality structure. However, due to the low sample sizes from crab emigration and lack of manipulative treatments, these findings remain tentative and should be researched further.

As variations in habitat quality become progressively more common from habitat fragmentation (Cushman, 2006; Lindenmayer & Fischer, 2013; Skole & Tucker, 1993), harvesting of natural resources (Beck et al., 2011; Lenihan & Peterson, 1998), and pollution (Fabricius, 2005; Li, Ma, van der Kuijp, Yuan & Huang, 2014), understanding how populations utilize spatially variable habitat will become increasingly important. The differences in mortality and dispersal that we have shown here demonstrate that population dynamics depends on personality and drastically differ across habitat quality. Furthermore, these personality‐driven differences have a high potential to mediate divergent community interactions and trophic cascades. Evaluating the personality composition of populations may therefore be an effective metric for predicting community responses to habitat degradation.

## CONFLICT OF INTEREST

None declared.

## AUTHOR CONTRIBUTIONS

BAB and BDG conceived and designed the experiments. BAB performed the experiments. BAB and BDG analyzed the data and wrote the manuscript.

## DATA ACCESSIBILITY

All relevant data files will be made available within the NSF online database URL: http://www.bco-dmo.org/project/562104 upon publication of this manuscript.
