# Supplementary material for: Personality interacts with habitat quality to govern individual mortality and dispersal patterns
Source: Ecol Evol. 2018 Jun 22;8(14):7216–27. doi: 10.1002/ece3.4257 (PMC6065346; doi:10.1002/ece3.4257)
Supplement: Supplementary file 1 [file ECE3-8-7216-s001.pdf]

Fig. S1.

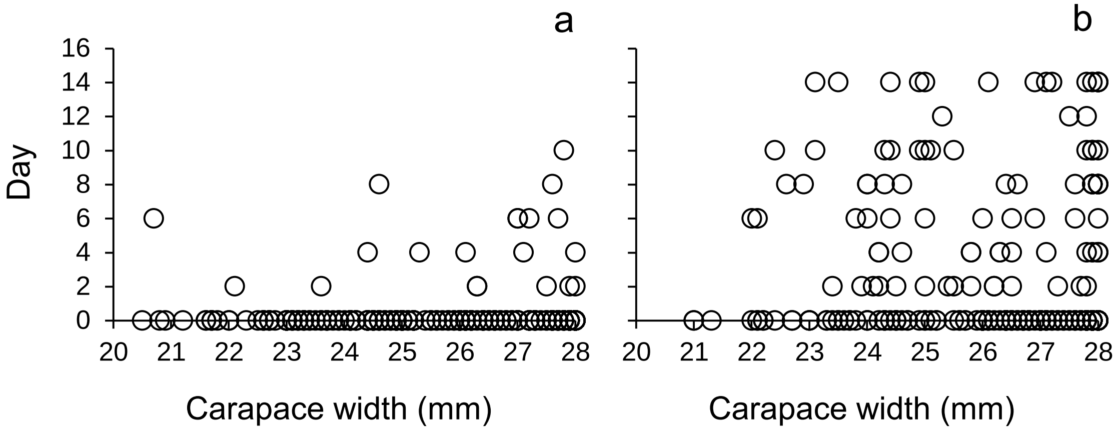


**Fig. S1.** Number of days crabs were found within 2.5 $m^2$ plots within **a)** low and **b)** high quality reefs (n = 240 crabs per reef type).
